# Supplementary material for: Terrestrial land cover shapes fish diversity in a major subtropical river catchment
Source: Commun Biol. 2025 Jul 28;8:1113. doi: 10.1038/s42003-025-08486-x (PMC12304115; doi:10.1038/s42003-025-08486-x)
Supplement: Supplementary file 3 — Reporting Summary [file 42003_2025_8486_MOESM3_ESM.pdf]

nature portfolio

Corresponding author(s): Florian Altermatt

Last updated by author(s): Mar 18, 2025

Reporting Summary

Nature Portfolio wishes to improve the reproducibility of the work that we publish. This form provides structure for consistency and transparency in reporting. For further information on Nature Portfolio policies, see our Editorial Policies and the Editorial Policy Checklist.

Statistics

For all statistical analyses, confirm that the following items are present in the figure legend, table legend, main text, or Methods section.

n/a	Confirmed	
☐	☒	The exact sample size (*n*) for each experimental group/condition, given as a discrete number and unit of measurement
☐	☒	A statement on whether measurements were taken from distinct samples or whether the same sample was measured repeatedly
☐	☒	The statistical test(s) used AND whether they are one- or two-sided<br>*Only common tests should be described solely by name; describe more complex techniques in the Methods section.*
☐	☒	A description of all covariates tested
☐	☒	A description of any assumptions or corrections, such as tests of normality and adjustment for multiple comparisons
☐	☒	A full description of the statistical parameters including central tendency (e.g. means) or other basic estimates (e.g. regression coefficient) AND variation (e.g. standard deviation) or associated estimates of uncertainty (e.g. confidence intervals)
☐	☒	For null hypothesis testing, the test statistic (e.g. *F*, *t*, *r*) with confidence intervals, effect sizes, degrees of freedom and *P* value noted<br>*Give P values as exact values whenever suitable.*
☒	☐	For Bayesian analysis, information on the choice of priors and Markov chain Monte Carlo settings
☒	☐	For hierarchical and complex designs, identification of the appropriate level for tests and full reporting of outcomes
☒	☐	Estimates of effect sizes (e.g. Cohen's *d*, Pearson's *r*), indicating how they were calculated

Our web collection on statistics for biologists contains articles on many of the points above.

Software and code

Policy information about availability of computer code

Data collection	Environmental DNA (eDNA) data in Thailand was collected by field work following well-established and standardized procedures. The flow direction map was provided by the HydroSHEDS database. Land cover data was provided by the European Space Agency Climate Change Initiative (ESA CCI) land cover map. Fish functional traits were collected from FISHMORPH database. Remote sensing (RS) data (Sentinel-2 Multispectral Instrument imagery) was preprocessed and downloaded from Google Earth Engine (GEE). All data collection was described in detail.
Data analysis	The catchment computing and FishDiv-LULC model-related analysis were programmed with Python and CUDA (for GPU computing). The RS imagery was preprocessed using peer-reviewed code available on the GEE. All code to reproduce data is available at Github (https://github.com/hengzhang-zh/FishDiv-LULC-Model).

For manuscripts utilizing custom algorithms or software that are central to the research but not yet described in published literature, software must be made available to editors and reviewers. We strongly encourage code deposition in a community repository (e.g. GitHub). See the Nature Portfolio guidelines for submitting code & software for further information.

Data

Policy information about availability of data

All manuscripts must include a data availability statement. This statement should provide the following information, where applicable:

- Accession codes, unique identifiers, or web links for publicly available datasets
- A description of any restrictions on data availability
- For clinical datasets or third party data, please ensure that the statement adheres to our policy

The code for FishDiv-LULC model can be found at Github (https://github.com/hengzhang-zh/FishDiv-LULC-Model). The eDNA sequencing data is publicly available on European Nucleotide Archive under tthe primary accession number PRJEB34331 (Kelly data set) and PRJEB34332 (MiFish data set).

Research involving human participants, their data, or biological material

Policy information about studies with human participants or human data. See also policy information about sex, gender (identity/presentation), and sexual orientation and race, ethnicity and racism.

Reporting on sex and gender	n/a
Reporting on race, ethnicity, or other socially relevant groupings	n/a
Population characteristics	n/a
Recruitment	n/a
Ethics oversight	n/a

Note that full information on the approval of the study protocol must also be provided in the manuscript.

Field-specific reporting

Please select the one below that is the best fit for your research. If you are not sure, read the appropriate sections before making your selection.

☐ Life sciences ☐ Behavioural & social sciences ☒ Ecological, evolutionary & environmental sciences

For a reference copy of the document with all sections, see nature.com/documents/nr-reporting-summary-flat.pdf

Ecological, evolutionary & environmental sciences study design

All studies must disclose on these points even when the disclosure is negative.

Study description	We developed a spatially explicit model to assess the spatial extent and magnitude of terrestrial land use and land cover (LULC) effects on fish diversity in a major river catchment in Thailand. Fish species richness derived from eDNA sampling was attributed to typical LULC types such as cropland, forest, and urban areas. Fish functional traits and river nutrient availability were regarded as the driving factor for LULC-fish species richness associations. We further projected fish diversity pattern with past and modeled future LULC using this model, and highlighted the necessity of LULC regulations.
Research sample	They are eDNA water samples from the major river channels in the Chao Phraya catchment in Thailand.
Sampling strategy	39 eDNA sampling sites representatively covered the whole catchment (see Fig. 1). For each site, six samples were collected from the left bank, channel center, and right bank (two replicates each). The field sampling was conducted during dry season of 2016 under base-flow conditions.
Data collection	eDNA samples were collected by Maslin Osathanunkul in 2016. Rosetta Blackman, Maslin Osathanunkul, Jeanine Brantschen, Cristina Di Muri, Lynsey Harper, and Bernd Hänfling did the bioinformatic analysis. The HydroSHEDS data was used to calculate catchment. Land cover data was provided by the ESA CCI land cover map. Fish functional traits were collected from FISHMORPH database. Sentinel-2 Multispectral Instrument imagery for river water properties was preprocessed and downloaded from GEE.
Timing and spatial scale	The Chao Phraya River catchment covers an area of 160,000 km2. The eDNA sampling campaign was conducted in November, 2016.
Data exclusions	Following conventional eDNA analysis approach, we removed 0.1% total reads for each sample to reduce uncertainties from processing.
Reproducibility	All the data analysis process can be reproduced, as both raw data and all code is available.

Randomization	n/a
Blinding	n/a

Did the study involve field work? ☒ Yes ☐ No

Field work, collection and transport

Field conditions	The average air and water temperatures during sampling were 30 °C and 26 °C, respectively. All field work was conducted under base-flow conditions in the dry season.
Location	The eDNA sampling was conducted in the Chao Phraya River catchment in Northern and Central Thailand. The elevation range of sampling sites is 2—509 m a.s.l.
Access & import/export	For each replicate, we sampled 100 mL using glass fiber filter membrane with 0.7 µm pore size (therefore, 600 ml for each sampling site). Then, samples were kept in a polystyrene box containing dry ice and stored at −20 °C until further processing. In addition, 300 mL ddH2O was filtered in the same manner for negative controls on each field day. We extracted all the samples within 2 days of sampling, following the standardized procedures (DNeasy Blood and Tissue Kit with a minor modification).
Disturbance	Sampling did not disturb the river system, all access was on publicly accessible sites.

Reporting for specific materials, systems and methods

We require information from authors about some types of materials, experimental systems and methods used in many studies. Here, indicate whether each material, system or method listed is relevant to your study. If you are not sure if a list item applies to your research, read the appropriate section before selecting a response.

Materials & experimental systems

n/a	Involved in the study	
☒	☐	Antibodies
☒	☐	Eukaryotic cell lines
☒	☐	Palaeontology and archaeology
☒	☐	Animals and other organisms
☒	☐	Clinical data
☒	☐	Dual use research of concern
☒	☐	Plants

Methods

n/a	Involved in the study	
☒	☐	ChIP-seq
☒	☐	Flow cytometry
☒	☐	MRI-based neuroimaging

Plants

Seed stocks	n/a
Novel plant genotypes	n/a
Authentication	n/a
